# The short-term and oncologic outcomes of younger VS older colorectal cancer patients undergoing primary surgery: a propensity score matching analysis

**DOI:** 10.1186/s12885-022-09246-4

**Published:** 2022-02-08

**Authors:** Xiao-Yu Liu, Bing Kang, Yu-Xi Cheng, Chao Yuan, Wei Tao, Bin Zhang, Zheng-Qiang Wei, Dong Peng

**Affiliations:** 1 Department of Gastrointestinal Surgery, the First Affiliated Hospital of Chongqing, Medical University, Chongqing, 400016 China; 2 Department of Clinical Nutrition, The First Affiliated Hospital of Chongqing Medical University, Chongqing, 400016 China

**Keywords:** Colorectal cancer, Younger, Older, Propensity score matching, Surgery

## Abstract

**Purpose:**

The purpose of the current study is to analyze the difference of short-term and oncologic outcomes between younger and older colorectal cancer (CRC) patients who underwent primary CRC surgery using a propensity score matching (PSM) analysis.

**Methods:**

We retrospectively collected CRC patients who underwent primary surgery in a single clinical database from Jan 2011 to Jan 2020. The short-term and oncologic outcomes were compared between younger aged group and older aged group.

**Results:**

A total of 4599 patients were included in this study, and there were 4196 patients in older aged group and 403 patients in younger aged group. After 1:1 ratio PSM, there were 401 patients in each group. No significant difference was found in terms of baseline information after PSM (p>0.05). Younger aged group had larger retrieved lymph nodes before (p<0.001) and after PSM (p=0.001) than older aged group. In multivariate analysis, younger age was an independent predictor of better overall survival (OS) (p<0.001, HR=2.303, 95% CI=1.658-3.199) and disease-free survival (DFS) (p=0.008, HR=1.425, 95% CI=1.098-1.850). In terms of different tumor stage after PSM, younger aged group had better OS than older group in stage II (p<0.001) and stage IV (p=0.028) CRC, and younger aged group had better DFS than older group in stage II (p=0.016) CRC.

**Conclusion:**

Younger CRC patients had larger retrieved lymph nodes and better prognosis than older CRC patients after primary CRC surgery.

## Introduction

Colorectal cancer (CRC) is one of the most common cancers with approximate 185 million cases worldwide [1], in addition, CRC is one of the most common causes of cancer-related deaths, with nearly 700,000 deaths every year [2]. Radical surgery is the cornerstone of CRC treatment [3–5].

The American Cancer Society updated the CRC screening guidelines recently. A major change was related to the start of screening age which was recommended from 50 to 45 years old [6]. Based on the recent data, although the incidence of CRC decreased, the incidence of younger CRC patients was increasing [7].

It remained controversial whether younger CRC patients were related to the prognosis [8–11]. Some studies reported that younger CRC patients had better prognosis [8], however, other studies reported younger CRC patients were associated with poorer prognosis [9–11]. Furthermore, there was only one study which analyzed the difference between younger and older CRC patients using a propensity score matching (PSM) analysis, however, this study focused on cancer-specific survival [12]. Therefore, the purpose of this study is to analyze the difference of short-term outcomes, overall survival (OS) and disease-free survival (DFS) between younger and older CRC patients who underwent primary CRC surgery using PSM.

## Methods

### Patients

We retrospectively collected CRC patients who underwent primary surgery in a single clinical database from Jan 2011 to Jan 2020. The study was approved by the ethics committee of our institution (The First Affiliated Hospital of Chongqing Medical University, 2021-517), and all patients signed informed consent forms. This study was conducted in accordance with the World Medical Association Declaration of Helsinki as well.

### Inclusion and exclusion criteria

Patients who underwent primary CRC surgery and diagnosed by pathology were included in this study (*n*=5473). The exclusion criteria were as follows: 1, Patients with incomplete clinical medical data (*n*=849); and 2, Non-R0 resection (n=25). Finally, a total of 4599 patients were included in this study.

### Surgery management and follow-up

The radical CRC surgery was according to the principles of oncology. Total mesorectal excision or complete mesocolic excision was performed, and the pathology confirmed R0 resection. Patients were followed up regularly three months for three years and six months for the following two years.

### Definitions

The tumor stage was diagnosed according to the AJCC 8^th^ Edition [13]. The younger aged group was defined as the age was ≤ 45 years old, the older aged group was defined as the age was > 45 years old. The complications were defined according to the Clavien-Dindo classification [14], and major complications were defined as ≥ III classification complications. OS was defined as the time from CRC surgery to death or last follow-up. DFS was defined as the time from CRC surgery to recurrence, death or last follow-up.

### Data collection

The perioperative and follow-up information were collected through the inpatient system, outpatient system and telephone interviews. The baseline information included sex, age, smoking, drinking, hypertension, type 2 diabetes mellitus (T2DM), coronary heart disease (CHD) and tumor location. The surgical information included operation time and blood loss. The pathologic information included tumor stage and retrieved lymph nodes. The postoperative information included complications and postoperative hospital stay.

### PSM

PSM was conducted between younger aged group and older aged group to minimize the bias of baseline information. Nearest neighbor matching was performed without replacement at a 1:1 ratio and a caliper width with a 0.01 standard deviation was specified. The matched baseline information was as follows: sex, BMI, drinking, smoking, T2DM, hypertension, CHD, tumor location and tumor stage.

### Statistical analysis

Continuous variables are expressed as the mean ± SD and independent-sample t test was used to analyze the difference between younger aged group and older aged group. Frequency variables are expressed as n (%), and Chi-square test or Fisher's exact test was used. The Kaplan-Meier curve was conducted to compare the age (younger/ older) on different tumor stage, and cox regression analyses were performed to identify independent predictive factors for OS and DFS. Data were analyzed using SPSS (version 22.0) statistical software. A bilateral *p* value of <0.05 was considered statistically significant.

## Results

### Patients

**Fig. 1:**
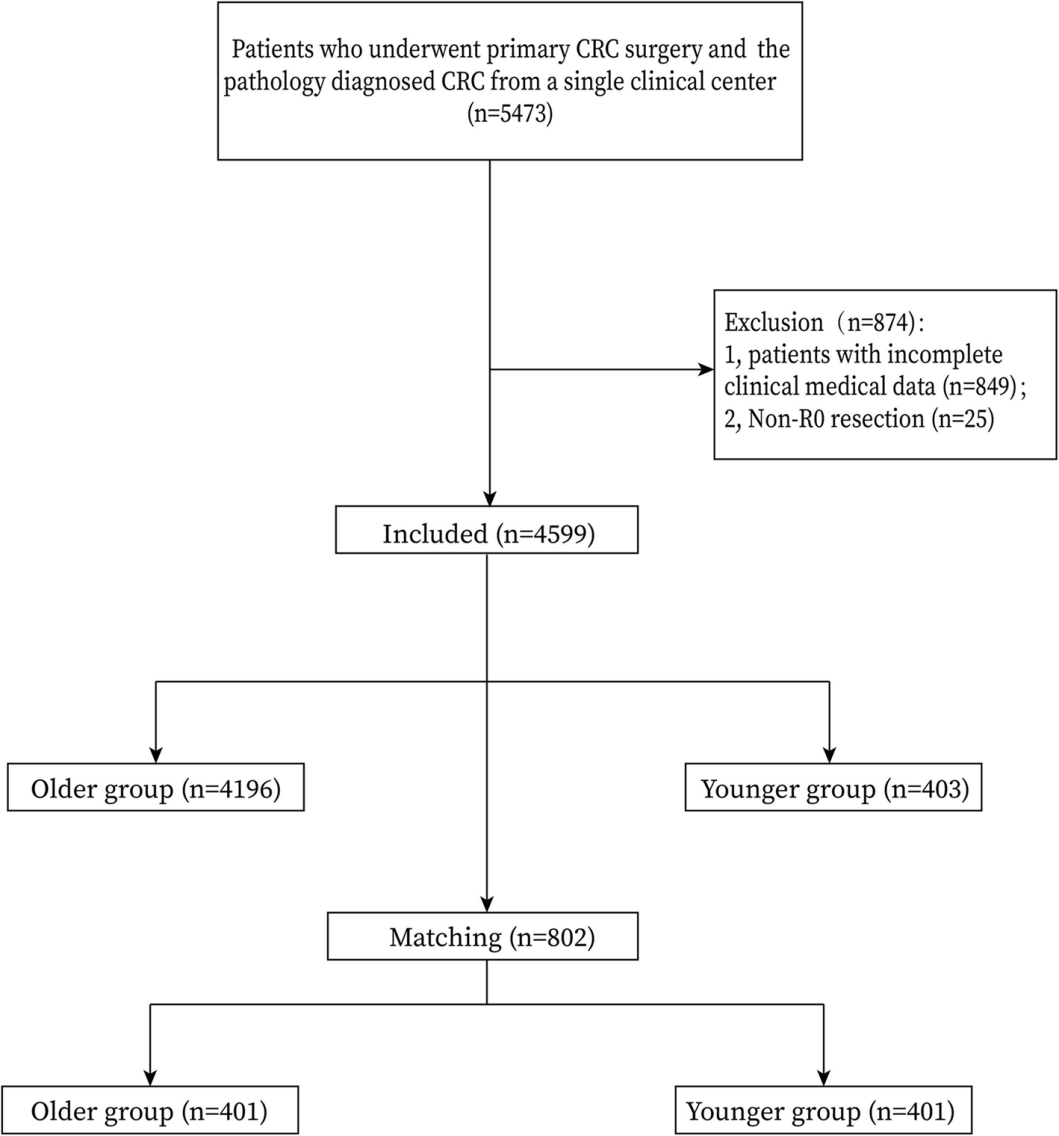
Flow chart of patient selection

A total of 4599 patients were included in this study according to the inclusion and exclusion criteria, and there were 4196 patients in older aged group and 403 patients in younger aged group. After 1:1 ratio PSM, there were 401 patients in each group (Fig 1).

### Baseline information

The baseline information was compared between the two groups. The age was 39.4 ± 5.4 years old in younger aged group and 65.1 ± 10.0 years old in older aged group. Younger group had higher portion of hypertension (*p*<0.001), T2DM (*p*<0.001) and CHD (*p*<0.001) before PSM. After 1:1 ratio PSM, there were 401 patients in each group, and no significant difference was found in terms of baseline information (*p*>0.05) (Table 1).

**Table 1:** Baseline characteristics before and after PSM

Characteristics	Before PSM			After PSM		
	Younger (403)	Older (4196)	*P* value	Younger (401)	Older (401)	*P* value
Age (year)	39.4 ± 5.4	65.1 ± 10.0	<0.001*	39.5 ± 5.4	63.2 ± 9.8	<0.001*
Sex			0.379			0.567
Male	229 (56.8%)	2479 (59.1%)		228 (56.9%)	236 (58.9%)	
Female	174 (43.2%)	1717 (40.9%)		173 (43.1%)	165 (41.1%)	
BMI (kg/m^2^)	22.8 ± 3.4	22.7 ± 3.2	0.323	22.8 ± 3.3	22.6 ± 3.2	0.325
Smoking	144 (35.7%)	1611 (38.4%)	0.293	143 (35.7%)	149 (37.2%)	0.660
Drinking	113 (28.0%)	1310 (31.2%)	0.187	112 (27.9%)	113 (28.2%)	0.937
Hypertension	22 (5.5%)	1169 (21.8%)	<0.001*	22 (5.5%)	24 (6.0%)	0.761
T2DM	7 (1.7%)	567 (13.5%)	<0.001*	7 (1.7%)	3 (0.7%)	0.203
CHD	1 (0.2%)	189 (4.5%)	<0.001*	1 (0.2%)	2 (0.5%)	1.000
Tumor location			0.235			0.724
Colon	201 (49.9%)	1963 (46.8%)		199 (49.6%)	194 (48.4%)	
Rectum	202 (50.1%)	2233 (53.2%)		202 (50.4%)	207 (51.6%)	
Tumor stage			0.988			0.947
I	75 (18.6%)	790 (18.8%)		75 (18.7%)	72 (18.0%)	
II	165 (40.9%)	1736 (41.4%)		165 (41.1%)	171 (42.6%)	
III	146 (36.3%)	1485 (35.4%)		144 (35.9%)	139 (34.7%)	
IV	17 (4.2%)	185 (4.4%)		17 (4.3%)	19 (4.7%)	

Note: Variables are expressed as the mean ± SD, n (%), *P-value <0.05.

Abbreviations: *T2DM* type 2 diabetes mellitus; *BMI* body mass index; *PSM* propensity score matching; *CHD* coronary heart disease.

### Short-term outcomes

The short-term outcomes included operation time, blood loss, retrieved lymph nodes, postoperative hospital stay and complications. We compared the difference between younger aged group and older aged group in terms of short-term outcomes. Younger aged group had larger retrieved lymph nodes before (*p*<0.001) and after PSM (*p*=0.001) than older aged group (Table 2).

**Table 2:** Short-term outcomes before and after PSM

Characteristics	Before PSM			After PSM		
	Younger (403)	Older (4196)	*P* value	Younger (401)	Older (401)	*P* value
Operation time (min)	236.8 ± 83.5	227.6 ± 83.5	0.038*	237.0 ± 83.6	227.9 ± 84.0	0.126
Blood loss (mL)	109.4 ± 127.3	106.9 ± 152.3	0.756	109.6 ± 127.6	105.5 ± 123.4	0.643
Retrieved lymph nodes	16.9 ± 8.8	14.6 ± 7.5	<0.001*	16.9 ± 8.8	15.0 ± 7.2	0.001*
Hospital stay (day)	11.3 ± 7.6	11.5 ± 8.8	0.682	11.3 ± 7.6	11.5 ± 8.2	0.731
Overall complications	77 (19.1%)	940 (22.4%)	0.128	76 (19.0%)	93 (23.2%)	0.141
Major complications	7 (1.7%)	104 (2.5%)	0.354	7 (1.7%)	14 (3.5%)	0.122

Note: Variables are expressed as the mean ± SD, n (%), *P-value <0.05

Abbreviations: *PSM* propensity score matching

### Univariate and multivariate analysis of OS and DFS

Univariate analysis was conducted to find potential factors for predicting OS and DFS, and multivariate analysis was conducted to identify independent predictors of OS and DFS.

In terms of OS, age (*p*<0.001, HR=2.303, 95% CI=1.658-3.199), tumor stage (*p*<0.001, HR=2.141, 95% CI=1.940-2.363), overall complications (*p*<0.001, HR=1.607, 95% CI=1.363-1.893) and major complications (*p*<0.001, HR=2.303, 95% CI=1.658-3.199) were independent predictors of OS (Table 3).

**Table 3:** Univariate and multivariate analysis of overall survival

Risk factors	Univariate analysis		Multivariate analysis	
	HR (95% CI)	*P* value	HR (95% CI)	*P* value
Age (older/ younger)	1.800 (1.313-2.468)	<0.001*	2.303 (1.658-3.199)	<0.001*
Sex (female/male)	0.865 (0.744-1.007)	0.061		
BMI (>/≤22.6)	0.842 (0.726-0.977)	0.023*	0.889 (0.766-1.032)	0.121
Hypertension (yes/no)	1.096 (0.927-1.294)	0.284		
T2DM (yes/no)	1.284 (1.037-1.589)	0.022*	1.136 (0.916-1.410)	0.245
Tumor site (colon/ rectum)	1.182 (1.020-1.370)	0.026	1.156 (0.997-1.340)	0.054
Tumor stage (IV/III/II/I)	2.138 (1.937-2.360)	<0.001*	2.141 (1.940-2.363)	<0.001*
Smoking (yes/no)	1.124 (0.968-1.306)	0.126		
Drinking (yes/no)	1.102 (0.942-1.290)	0.226		
CHD (yes/no)	1.338 (0.943-1.899)	0.102		
Overall complications (yes/no)	1.807 (1.548-2.108)	<0.001*	1.607 (1.363-1.893)	<0.001*
Major complications (yes/no)	2.900 (2.130-3.948)	<0.001*	2.303 (1.658-3.199)	<0.001*

Note: *P-value <0.05

Abbreviations: *HR* Hazard ratio; *CI* confidence interval; *BMI* body mass index; *T2DM* type 2 diabetes mellitus; *CHD* coronary heart disease

As for DFS, age (p=0.008, HR=1.425, 95% CI=1.098-1.850), tumor stage (*p*<0.001, HR=2.100, 95% CI=1.921-2.295), overall complications (*p*<0.001, HR=1.504, 95% CI=1.293-1.750) and major complications (*p*<0.001, HR=2.015, 95% CI=1.461-2.776) were independent predictors of DFS (Table 4).

**Table 4:** Univariate and multivariate analysis of disease-free survival

Risk factors	Univariate analysis		Multivariate analysis	
	HR (95% CI)	*P* value	HR (95% CI)	*P* value
Age (older/ younger)	1.405 (1.083-1.823)	0.011*	1.425 (1.098-1.850)	0.008*
Sex (male/female)	0.877 (0.764-1.006)	0.062		
BMI (>/≤22.6 kg/m^2^)	0.889 (0.777-1.017)	0.086		
Hypertension (yes/no)	1.064 (0.914-1.239)	0.424		
T2DM (yes/no)	1.117 (0.913-1.367)	0.281		
Tumor site (colon/ rectum)	1.113 (0.973-1.273)	0.118		
Tumor stage (IV/III/II/I)	2.090 (1.911-2.285)	<0.001*	2.100 (1.921-2.295)	<0.001*
Smoking (yes/no)	1.114 (0.972-1.276)	0.122		
Drinking (yes/no)	1.115 (0.966-1.286)	0.137		
CHD (yes/no)	1.263 (0.916-1.742)	0.155		
Overall complications (yes/no)	1.644 (1.425-1.897)	<0.001*	1.504 (1.293-1.750)	<0.001*
Major complications (yes/no)	2.451 (1.809-3.319)	<0.001*	2.015 (1.461-2.776)	<0.001*

Note: *P-value <0.05

Abbreviations: *HR* Hazard ratio; *CI* confidence interval; *BMI* body mass index; *T2DM* type 2 diabetes mellitus; *CHD* coronary heart disease

### The effect of younger aged group and older aged group on different tumor stages

**Fig. 2:**
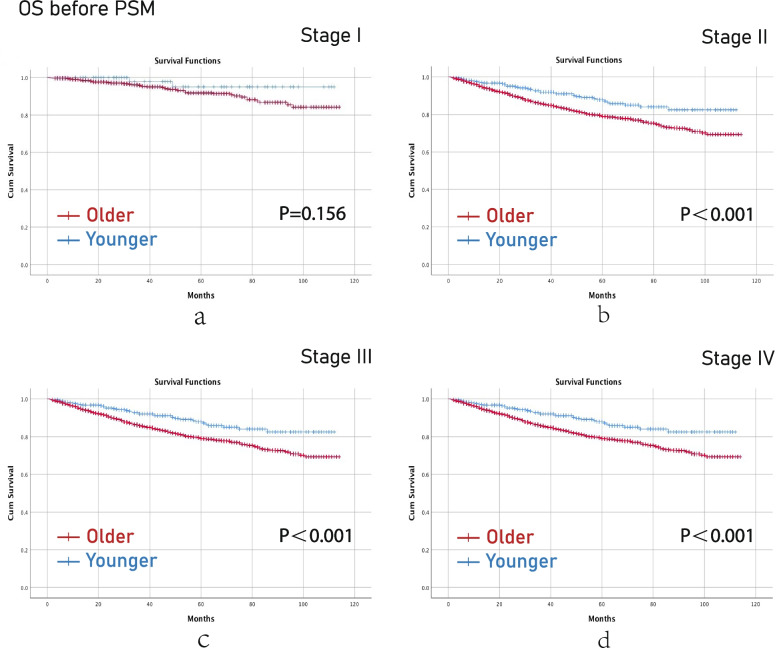
OS before PSM. (**a**), stage I; (**b**), stage II; (**c**), stage III; (**d**), stage IV. Note: OS, overall survival; PSM, propensity score matching

**Fig. 3:**
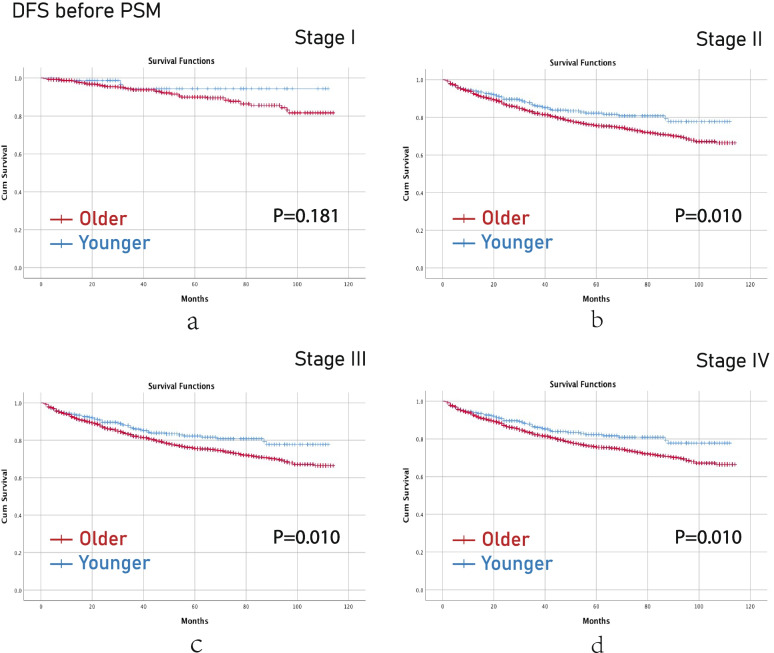
DFS before PSM. (**a**), stage I; (**b**), stage II; (**c**), stage III; (**d**), stage IV. Note: DFS, disease-free survival; PSM, propensity score matching

Before PSM, younger aged group had better OS than older aged group in terms of stage II (*p*<0.001), stage III (*p*<0.001) and stage IV (*p*<0.001) CRC (Fig 2). Younger aged group had better DFS than older aged group in terms of stage II (p=0.010), stage III (*p*=0.010) and stage IV (*p*=0.010) CRC as well (Fig 3).

**Fig. 4:**
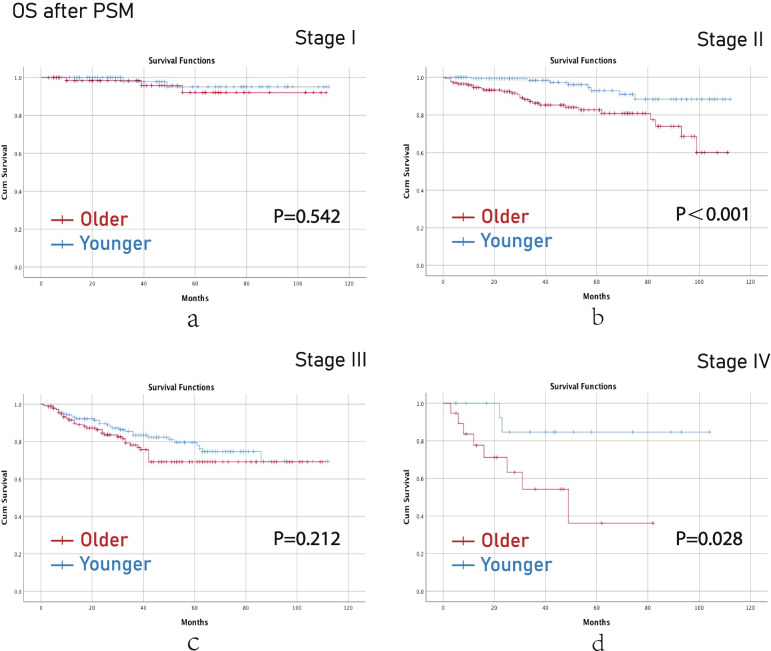
OS after PSM. (**a**), stage I; (**b**), stage II; (**c**), stage III; (**d**), stage IV. Note: OS, overall survival; PSM, propensity score matching

**Fig. 5:**
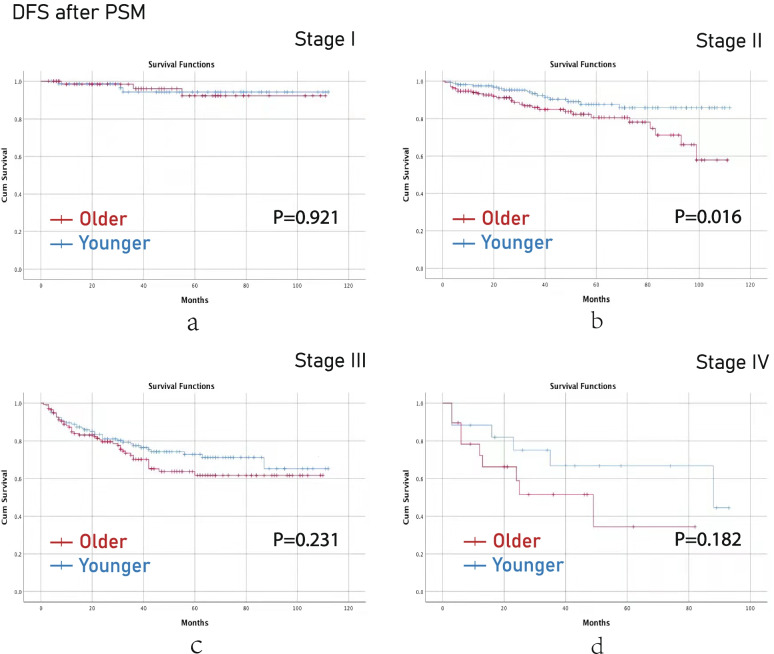
DFS after PSM. (**a**), stage I; (**b**), stage II; (**c**), stage III; (**d**), stage IV. Note: DFS, disease-free survival; PSM, propensity score matching

After PSM, younger aged group had better OS than older aged group in terms of stage II (*p*<0.001) and stage IV (*p*=0.028) CRC. (Fig 4) Younger aged group had better DFS than older aged group in terms of stage II (*p*=0.016) CRC (Fig 5).

## Discussion

A total of 4599 patients were included in this study and there were 4196 patients in older aged group and 403 patients in younger aged group. After 1:1 ratio PSM, there were 401 patients in each group. No significant difference was found in terms of baseline information after PSM. Younger aged group had larger retrieved lymph nodes before and after PSM. In multivariate analysis, younger age was an independent predictor of better OS and DFS. In terms of different tumor stages after PSM, younger aged group had better OS than older aged group in stage II and stage IV CRC, and younger aged group had better DFS than older aged group in stage II CRC.

The impact of age on the prognosis of CRC was still controversial [8–11], Zhao L [9]. et al reported a poor prognosis for CRC in younger patients, but Yang Z [15]. et al reported that the prognosis of younger patients was similar to that of older patients. However, another study reported a better prognosis for younger patients [8]. We summarized the previous studies which reported the age on the outcomes of CRC patients in Table 5 [8–12, 15–18]. As was shown in the table, OS, DFS, CSS (cancer-specific survival) and short-term outcomes were the main indicators. Moreover, short-term outcomes were reported in two studies, which were relatively small [12, 17]. Furthermore, no previous studies reported the age on the survival outcomes of specific tumor stages. PSM analysis was method to reduce the selection bias of the baseline information, which could benefit precise results when there was no difference in baseline information [19, 20]. Therefore, the current study aimed to explore the specific impact of age on CRC including short-term outcomes, OS and DFS in different tumor stages using PSM.

**Table 5:** Previous studies reporting the age on the outcomes of CRC patients

Author	Year	Country	Sample size	Cut-off age (years)	Younger group	Older group	Patients	Outcomes
Nakayama Y [8]	2020	Japan	5602	40	359	5243	Stage II-III CRC	OS, DFS
Zhao L [9]	2017	China	995	35	68	927	Stage I-III CRC	OS, DFS, CSS
Fu JF [10]	2013	China	1335	30	42	1239	Stage I-IV CRC	OS
Shida D[11]	2018	Canada	861	40	66	795	Stage IV CRC	OS
Wang L [12]	2020	Japan	3095	45	139	2956	Stage I-III CRC	DFS, CSS, Short-term outcomes (Using PSM)
Yang Z [15]	2012	Japan	3156	45	530	2626	Stage I-IV CRC	OS, DFS, CSS
Quah HM [16]	2007	USA	1327	40	68	1259	Stage I-III CC	OS, CSS
Schellerer VS [17]	2012	Germany	1962	50	244	1718	Stage I-IV CRC	OS, CSS, Short-term outcomes
Wong SW [18]	2021	Malaysia	2127	50	206	1921	Stage I-IV CRC	OS, CSS

Abbreviations: *CRC* colorectal cancer; *CC* colon cancer; *OS* overall survival; *DFS* disease-free survival; *CSS* cancer-specific survival; *PSM* propensity score matching

However, the cut-off age of younger CRC patients was different in previous studies, and the cut-off age included 30, 35, 40, 45 and 50 years old [8–12, 15–18]. In this study, we used 45 years old as the cut-off of younger and older CRC patients, which was according to the recommended screening age and previous studies [6, 8, 12, 15]. In addition, PSM was used in this study to analyze the different outcomes between younger aged group and older aged group.

There were many factors that could affect the prognosis of CRC patients including tumor stage, BMI, T2DM, age and complications [3, 21–24]. In this study, tumor stage and complications were independent predictors of CRC patients which was similar with previous studies [3, 21, 22]. In addition, younger aged patients were associated with better prognosis than older aged patients, which meant that after radical CRC surgery, age was an important factor affecting the prognosis. Therefore, in order to explore the effect of age on each specific tumor stage, Kaplan-Meier was used and we found that younger aged group had better OS than older aged group in stage II and stage IV CRC, and younger aged group had better DFS than older aged group in stage II CRC. The mechanism was unclear, and it was found in a previous study that younger patients had better prognosis in stage III CRC [12], so more researches are needed on specific tumor stage in the future.

In addition to prognosis, previous studies rarely reported age on short-term outcomes. Only one study reported the shot-term outcomes of after CRC surgery [12]. Wang L [12]. et al reported that there was no difference between younger aged group and older aged group, however, the information of retrieved lymph nodes was missing and they failed to match the baseline information. In this study, younger CRC patients had more retrieved lymph nodes than older CRC patients. The possible reasons might be as follows: First, younger CRC patients might have better anatomy, which was more convenient to harvest lymph nodes; Second, surgeons might be more inclined to operate on younger CRC patients with a larger range. Furthermore, less retrieved lymph nodes might contribute to poor prognosis in older CRC patients [25–27].

To our knowledge, this study analyzed the short-term outcomes of younger and older CRC patients with the largest amount of data and compared the retrieved lymph nodes between younger and older CRC patients for the first time. Furthermore, PSM was used, OS and DFS were compared for specific tumor stage. However, some limitations exited in this study. First, this was a retrospective single center study; Second the follow-up time was relatively short; Third, the definition of younger age was not the same in previous studies, we chose the cut-off of 45 years old based on the recommended CRC screening age and published studies. Thus, multi-center prospective randomized controlled trials with comprehensive perioperative information should be performed in the future.

In conclusion, younger CRC patients had larger retrieved lymph nodes and better prognosis than older CRC patients after primary CRC surgery.

## Data Availability

The datasets used and analyzed during the current study are available from the corresponding author on reasonable request.
